# Walking Paths to and from a Goal Differ: On the Role of Bearing Angle in the Formation of Human Locomotion Paths

**DOI:** 10.1371/journal.pone.0121714

**Published:** 2015-04-10

**Authors:** Manish Sreenivasa, Katja Mombaur, Jean-Paul Laumond

**Affiliations:** 1 Optimization in Robotics and Biomechanics, Interdisciplinary Center for Scientific Computing, University of Heidelberg, Heidelberg, Germany; 2 LAAS-CNRS, University of Toulouse, Toulouse, France; University of Utah, UNITED STATES

## Abstract

The path that humans take while walking to a goal is the result of a cognitive process modulated by the perception of the environment and physiological constraints. The path shape and timing implicitly embeds aspects of the architecture behind this process. Here, locomotion paths were investigated during a simple task of walking to and from a goal, by looking at the evolution of the position of the human on a horizontal (x,y) plane. We found that the path while walking to a goal was not the same as that while returning from it. Forward-return paths were systematically separated by 0.5-1.9m, or about 5% of the goal distance. We show that this path separation occurs as a consequence of anticipating the desired body orientation at the goal while keeping the target in view. The magnitude of this separation was strongly influenced by the bearing angle (difference between body orientation and angle to goal) and the final orientation imposed at the goal. This phenomenon highlights the impact of a trade-off between a directional perceptual apparatus—eyes in the head on the shoulders—and and physiological limitations, in the formation of human locomotion paths. Our results give an insight into the influence of environmental and perceptual variables on human locomotion and provide a basis for further mathematical study of these mechanisms.

## Introduction

Locomotion, in the simplest of terms, allows for a displacement from one place to another. Humans and other animals move to reach a goal (an office to go to or to forage for food), and this intentionality implicitly embeds the role of cognition, perception and biomechanics into the shape and timing of the locomotion path. We may therefore study the path to investigate these underlying processes. Typically, participants are recorded while walking from an origin to a goal in uncluttered space [1–5], or in the presence of obstacles/dynamic environments [6–8]. The walking conditions are modulated by varying visual conditions [3, 9], goal configurations [1, 4] or turning characteristics [2, 10, 11] and their influence on the path studied to better understand the underlying mechanisms (see e.g. [12] for an overview). Taken together, the results from numerous studies show that although the human locomotor system is highly redundant (i.e. there are a theoretical infinite ways to go from A to B), the actual paths taken are highly systematic. Models that can reproduce this predictability may use geometry-based feedback laws [7, 8, 13, 14], a combination of open-loop and feedback processes [3], or optimization frameworks [4, 15].

**Fig 1:**
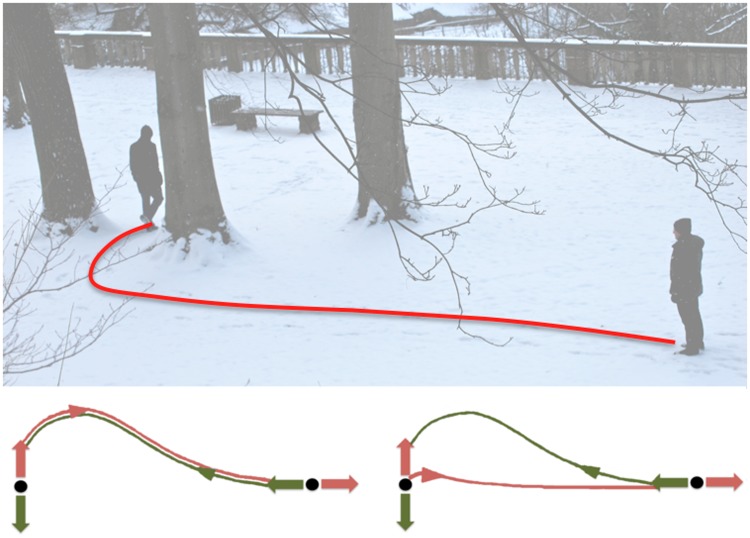
Traces of locomotion. The path that humans take to walk to a goal is modulated not only by the goal position but also the final body orientation desired. The effects of this modulation can be deduced for example by looking at the trace of the walking path on the ground. Bottom panels illustrate two scenarios for paths to and from a goal. Human locomotion paths exhibit the kind of separated paths shown in the bottom right panel. This study investigates the underlying causes behind this observation.

The focus of the current study is in the context of such locomotion models that define the path taken by humans to go from a starting posture with position (*x*_1_, *y*_1_) and orientation *θ*_1_, to a goal posture with position (*x*_2_, *y*_2_) and orientation *θ*_2_, Fig 1. The choice of the reference frame for the orientation *θ* is crucial, and reveals a special property of human locomotion. Studies looking at goal directed locomotion in humans have found that when walking from a starting posture to a goal posture, the human body behaves as a *non-holonomic* system most of the time [16]. Non-holonomy is defined as the property of a moving agent where its orientation is identical to its heading *ρ* (direction of locomotion calculated as the tangent to successive positions). This is a well-grounded research topic in mobile robotics [17]. Non-holonomic movements are for example the smooth paths executed by a car. In contrast, holonomic movements are executed by a decoupling between orientation and heading, such as the crab-like motions we may use to avoid people in a highly crowded environment. Outside of these special conditions, the non-holonomic nature of human locomotion allows us to infer the body orientation from heading information [16].

If we just consider the variables (*x*, *y*, *θ*), the shortest path from (*x*_1_, *y*_1_, *θ*_1_) to (*x*_2_, *y*_2_, *θ*_2_) should be identical to that from (*x*_2_, *y*_2_, −*θ*_2_) to (*x*_1_, *y*_1_, −*θ*_1_). From a mathematical point of view, this is a straightforward consequence of the invariance of trajectories by isometric transformations [17]. This is typically the case for the paths taken by a car. However, observations from the study by Mombaur et al. [4] suggest that human locomotion violates this symmetry property, i.e. the path when walking from an origin to a goal was not the same as that from the goal to the origin, Fig 1. In that study, the separation between forward-return paths was attributed to the bearing angle (angle between the body direction and the angle to goal) and linked to perceptual rather than mechanical causes.

The current study methodically investigates this separation between forward and return paths during goal-directed walking. We hypothesized that the observed differences are due to a perceptuo-motor trade-off between goal anticipation and physiological constraints. To test this hypothesis we designed three experiments where participants had to walk under varying conditions of goal positions and orientations. As part of our investigation, we answer the following questions successively: Is there a systematic separation between forward and return locomotion paths? If yes, how is this separation affected by goal bearing, and goal distance?

## Materials and Methods

### Participants

Walking trajectories of 18 participants were recorded with the following demographics, Mean ± SD: age 26.3 ± 2.8 years, weight 65.7 ± 9.7 kg, height 1.7 ± 0.09 m. 10 participants took part in Experiment 1 (7 males, 3 females), 8 in Experiment 2 (4 males, 4 females, with 2 common participants with Experiment 1) and 5 in Experiment 3 (all males, with 2 common participants with Experiments 1 and 2). Participants volunteered to take part in the experiment, and were recruited by convenience sampling from a pool of about 300–400 researchers at LAAS-CNRS, Toulouse, France. Volunteers that were familiar with the study and its background were excluded (e.g. colleagues from the same working group), in order to maintain naivety about the expected behavior. Participants had normal or corrected-to-normal vision and reported to be in good general health. Before start of recordings, the experimental protocol was explained to each participant verbally and by practising a few representative trials. Written consent was obtained by asking the participants to report their age, weight and height on a preformatted document, and sign their consent to usage of the recorded data. The experiments were conducted in accordance with the standards of the Declaration of Helsinki (rev. 2013), with formal approval of the ethics evaluation committee Comité d’Evaluation Ethique de l’Inserm (IRB00003888, Opinion number 13-124) of the Institut National de la Santé et de la Recherche Médicale, INSERM, Paris, France (IORG0003254, FWA00005831).

### Apparatus

Walking trajectories of the participants were recorded using a Global Positioning System (GPS) instrumented backpack in all experiments. The high accuracy required to capture trunk position was achieved by using a Carrier-Phase Differential GPS setup, C-DGPS. C-DGPS utilizes a secondary static GPS unit (master station) to correct for errors in a mobile rover GPS in its vicinity. The rover GPS used in this experiment was a Novatel Propak V3 L1 receiver in combination with an aviation grade, light (< 200 g) and compact Novatel GPS 532 antenna. The antenna was mounted onto a short pole, which was fixed to a frame inside a backpack. Data were output at 20 Hz with a typical position deviation of 1cm depending on quality of satellite data and environmental conditions such as tree cover, reflections etc. Master station data was recorded from a static GPS unit (Novatel Propak-G1 Plus, Novatel—GPS 702 antenna). The GPS receiver, data logger and battery pack were carried in the backpack altogether weighing about 4.4 kg. A hand-held push button was provided to the participants in order to record the start and stop of walking as a series of trigger points on the recorded timeline.

### Procedures

Experiments 1 and 2 were conducted outdoors in a large, 20×15m, empty flat area with a tarmac surface. Experiment 3 was conducted in a flat grass field with an approximate size of 50×100m.

#### Experiment 1: Posture-to-Posture Walking (PtP)

**Fig 2:**
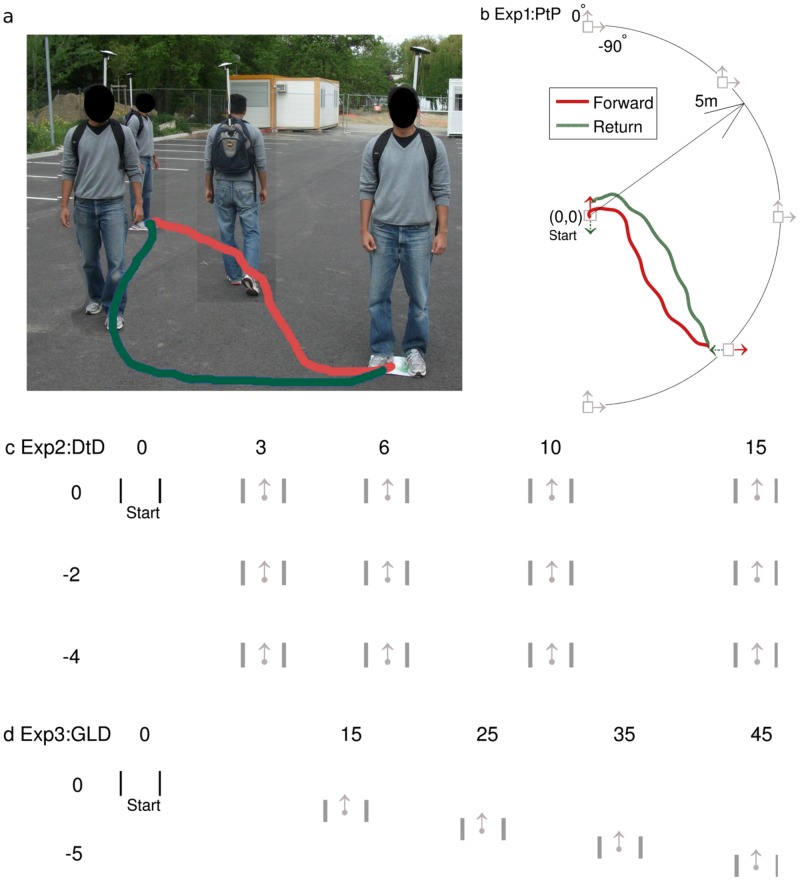
Experimental layouts. (A) Superimposed pictures illustrate the posture of the participants during the start, middle and end of the forward and return paths. (B) Exp 1, PtP—Fixed starting position (0,0), orientation (0°, straight ahead), and various goal positions and orientations are indicated by the squares and arrows. Sample forward (solid line) and return (dashed line) paths are shown for the (GA = −135°, GO = −90°) condition. (C) Exp 2, DtD—Start and goal positions were set in a rectangular grid. Note that participants started and stopped 2m before and after the doors. (D) Exp3, GLD—Goals were located at a fixed angle of −108° and at varying distances.

Participants were asked to walk from a starting position and orientation to a goal position and orientation, stop, turn around, and walk back to the starting position such that their final orientation was rotated by 180° relative to the starting orientation. The experiment followed a: 5 Goal Angle, GA [0°, −45°, −90°, −135°, −180°] × 2 Goal Orientation, GO [0°, −90°] design, Fig 2b. The layout of this experiment was similar to that in Mombaur et al. [4]. The major modification was that here we specifically tested pairs of forward-return paths as a function of the goal angle. We henceforth denote the first part of the walking path (Start1 to Goal1) as the forward path, and the second part (Start2 to Goal2) as the return path. Fig 2a and 2b illustrates sample forward and return paths for the (GA = −135°, GO = −90°) condition. Note that GA and GO were defined as clockwise negative relative to the starting configuration of the forward path. Based on this layout, all conditions with GO = 0° resulted in isometric forward-return conditions. This means that at the start of both the forward and return paths the goal lay at the same bearing angle. For all conditions with GO = −90°, the forward-return conditions were anisometric with a 90° shift between the goal angle of the forward path and that of the return path.

Start and goal positions were indicated by flat cardboard squares (0.3 × 0.3*m*) with the orientations drawn on them as large visible arrows. Goal positions were located at a radius of 5m from the starting position. Trials began with participants standing at the start position (0, 0) facing the forward direction (0°) as illustrated in Fig 2a and 2b. An experimenter moved a second cardboard square to the goal position and placed it in the required orientation. Before starting to walk, participants were asked to make sure they knew where the goal lay by looking at it. Participants pressed the hand-held button to indicate start of walking and started walking towards the goal. They stopped at the goal position in the orientation indicated, and pressed the button a second time to indicate the end of walking. They then paused for 2 seconds, turned around by 180°, paused for another 2 seconds, and repeated the procedure while walking back to the start position. Note that the return path goal orientation was rotated by 180° relative to the forward path starting orientation.

Participants practised a random sample of trials for about 5 minutes to familiarize themselves with the protocol and apparatus. They were asked to start and stop walking inside the areas indicated by the cardboard squares and to orient their body, in the beginning and end of walking, as close as possible to the indicated orientations. There were no further instructions given about the walking behavior. Each GA x GO pair was tested twice, giving a total of 20 forward-return paths per participant. Trials were presented in a randomized order and for each participant the entire experiment was conducted in a single session of about 1 hour.

#### Experiment 2: Door-to-Door Walking (DtD)

Experiment 2 was designed with the same forward-return protocol as Experiment 1. In addition, we introduced the following modifications to the walking conditions:

- The start and goal orientations were defined by doors that participants had to walk through
- Participants were asked to start walking 2m behind the start door, and stop 2m after crossing the goal door
- A variation of goal distances were tested

Fig 2c shows the layout of the DtD experiment and the various combinations of goal positions. In contrast to the PtP protocol, all goals were located to the side and behind the starting position with respect to the starting orientation. We opted for a rectangular grid arrangement of goal positions, due to the unavailability of an experimental area large enough to accommodate a circular grid at these goal distances. The rectangular grid consisted of 4 Lateral Distance, LD [3,6,10,15m] × 3 Sagittal Distance, SD [0,-2,-4m]. We measured 3 goal orientations, GO = 0°, −45° and −90°. We also measured trials where participants started and stopped between the doors (as opposed to 2m before and after the doors). However, for the purpose of our present analysis we only consider the trials with isometric orientation, i.e. GO = 0°, and with starts and stops 2m before and after the doors. The analysis of the additional recorded trajectories will be the focus of a future study.

Participants were asked to walk between two doors, characterized by identical pairs of large vertical boxes with the dimensions 1.0×0.3×0.3m spaced 1m apart. Each LD x SD pair was tested once to give a total of 12 forward-return paths per participant. Trials were presented in a randomized order and for each participant the experiment was conducted in a single session of about 1 hour.

#### Experiment 3: Goals at Large Distances (GLD)

Similar to the DtD experiment, participants were asked to walk from one door to another located at varying distances. Goal positions were located at a constant GA of 108° and GO of 0°. The lateral distances recorded were 15, 25, 35 and 45m. Fig 2d plots the experimental layout. Other experimental conditions were identical to the DtD protocol. Each goal distance was recorded twice, in a single experimental session of about 30 minutes per participant.

### Data Analysis

Raw GPS walking data were processed in the C-DGPS software GrafNav 7.6 (Novatel Inc., USA). GPS position data were output in the local Universal Transverse Mercator (UTM) Zone 31 projection (WGS84 datum). The software used to process GPS data provided instantaneous localization error estimates in the local X, Y and Z coordinates. Data were verified to ensure that the positioning errors were below a threshold of 1cm. A more detailed discussion of C-DGPS localization is available in the study by Luo & Lachapelle [18] and in Terrier et al. [19], Frissen et al. [20] for its the application in human locomotion studies. Data were transformed such that the new coordinate frame was centered at the starting position with the positive y-axis parallel to the forward orientation in the trials (0° in Fig 2b). Further data processing was conducted in Matlab 2013a (Matlab Inc., USA). The push-button trigger events were used to automatically segment position data into trials. In 6 trials (from a total of 336) participants mistakenly pressed the trigger button, these trials were removed from further analysis. Segmented position data were filtered using a 3rd order low-pass Butterworth filter with a threshold frequency of 0.8 Hz. The filter frequency was chosen such that it removed oscillations due to stepping. We used a symmetric forward-backward pass filter such that no phase delay was introduced in the data. Filtered paths were then resampled such that each pair of forward-return paths consisted of the same number of data points.

The separation between forward and return paths were quantified by the measure, mean Path Separation (mPS), calculated as:

$$\begin{array}{c} mPS=\frac{1}{N}\sum_{\tau =0}^{1}\sqrt{{\left({\vec{X}}_{\tau }-{\overset{\leftarrow }{X}}_{1-\tau }\right)}^{2}+{\left({\vec{Y}}_{\tau }-{\overset{\leftarrow }{Y}}_{1-\tau }\right)}^{2}} \end{array}$$ (1)

where, $\left(\vec{X},\vec{Y}\right)$ and $\left(\overset{\leftarrow }{X},\overset{\leftarrow }{Y}\right)$ were the recorded positions of the forward and return paths, respectively. *τ* denotes the normalized time epoch ranging from 0 to 1, and N the total number of frames. Note that as the paths were resampled, each forward-return pair consisted of the same number of frames, N. As a consequence mPS equals zero if the paths were identical. We computed the path-length normalized separation measure as:

$$\begin{array}{c} WmPS=\frac{mPS}{d} \end{array}$$ (2)

where, d = distance to goal. Recall that in the PtP trials the starting and goal positions were indicated by cardboard squares. In our analysis, we only considered the path between these squares, i.e. outside the [0.3 × 0.3*m*] area, to avoid artefacts due to delays in pressing the trigger and body sway at standstill. Similarly for the DtD and GLD trials, we only considered the path between the start and goal doors to calculate the separation measures. Heading *ρ*, was calculated as the instantaneous direction of walking from successive recorded (x,y) positions. Instantaneous bearing Ψ, was calculated as the difference between the heading *ρ*, and the angle to goal, *β*:

$$\begin{array}{c} \Psi (\tau )=\rho (\tau )-\beta (\tau ) \end{array}$$ (3)

**Fig 3:**
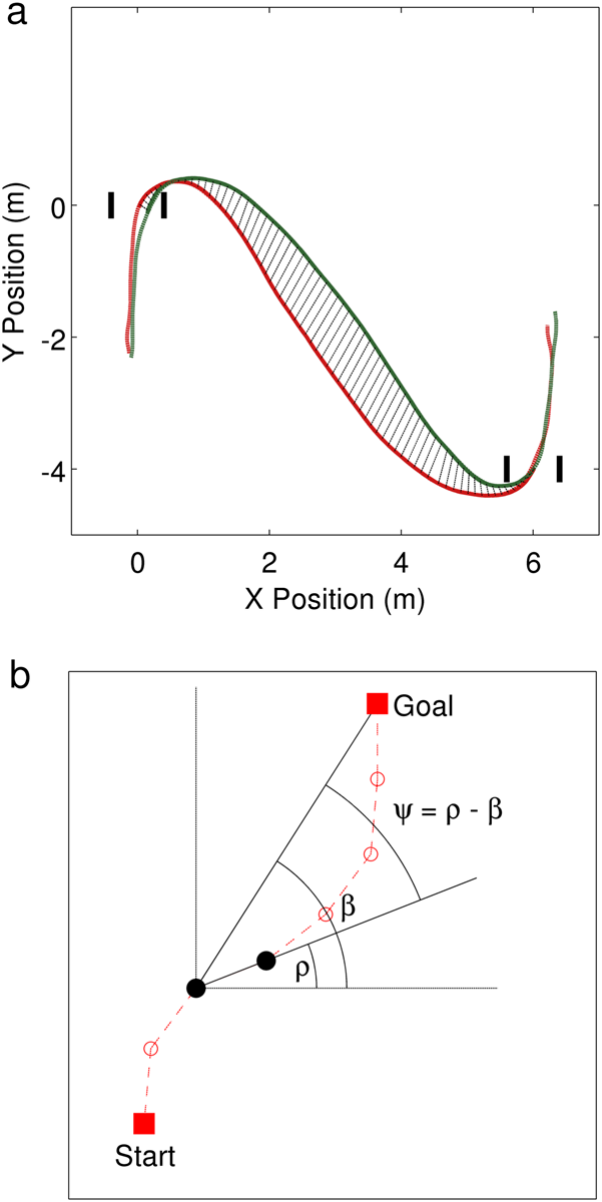
Data analysis. (A) Mean separation between forward-return paths was computed from the recorded positions. Lines between the forward-return paths indicate the time-matched epochs, *τ* on the forward path and 1 − *τ* on the return path. (B) Heading *ρ*, angle to target *β*, and bearing Ψ, were calculated from successive positions recorded along the path (dashed red line through open circles).

Fig 3 illustrates these measures. For the DtD and GLD experiments we also computed a term Ψ^*overshoot*^, which quantified the mean overshoot of bearing angle. As participants turned towards the goal at the start of walking, the heading *ρ* converged towards the angle to goal *β*, i.e. bearing angle Ψ tended to zero. We define the first instance where Ψ crosses zero as the *crossover point*. The measure Ψ^*overshoot*^ was calculated as the mean bearing angle after this crossover point, until the end of walking.

## Results

The statistical tests reported here were conducted in SPSS 21.0 (IBM Corp., USA). Significance level was set at a p-value of 0.05. Mauchly’s test was used to confirm the assumption of sphericity, and in cases of violation we applied the Greenhouse-Geisser correction and report the corrected degrees of freedom.

### Systematic separation in forward-return paths

**Fig 4:**
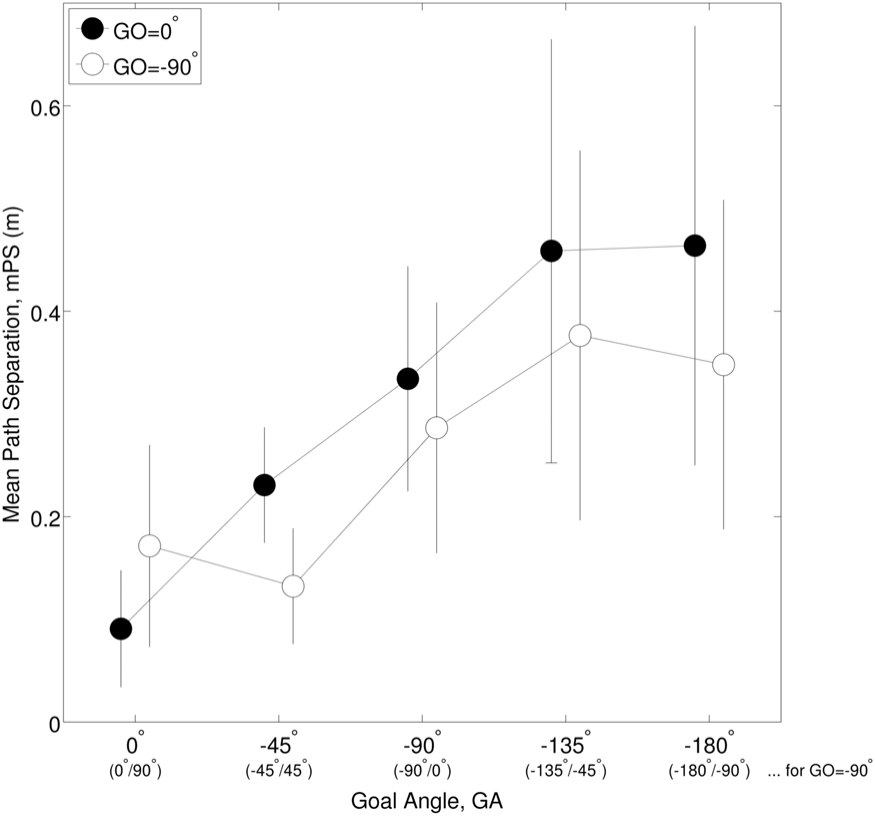
Mean path separation (mPS)—PtP trials. mPS is plotted as a function of goal angle, GA. The labels for x-axis in brackets indicate the anisometric forward-return goal angles for the GO = −90° condition. Error bars indicate standard deviation of the mean over all participants.

Fig 4 plots the effect of goal angle on the path separation measure, mPS. Path separation monotonically increased from 0.1312m for GA = 0°, to, 0.4060m for GA = −180°. Note that for the combination GA = 0° and GO = 0°, the walking task involved walking straight to a target directly in front and back. It is likely that the path separation observed in these trials (0.1312m) was due to variability in stepping and measurement noise, rather than the perceptual related mechanisms being studied here. We observed that paths for the anisometric conditions (GO = −90°) showed consistently smaller separation than the corresponding isometric conditions (except for GA = 0°). We found a significant main effect of GA, F(4,36) = 12.316, p < 0.001, *η* ^2^ = 0.578, with no higher order GAxGO interaction effect p = 0.074. mPS measures were subsequently collapsed across the GO condition. Pairwise contrast tests showed that the GA pairs [0°, −45°] and [−45°, −90°] were significantly different.

### Effect of goal distance

**Fig 5:**
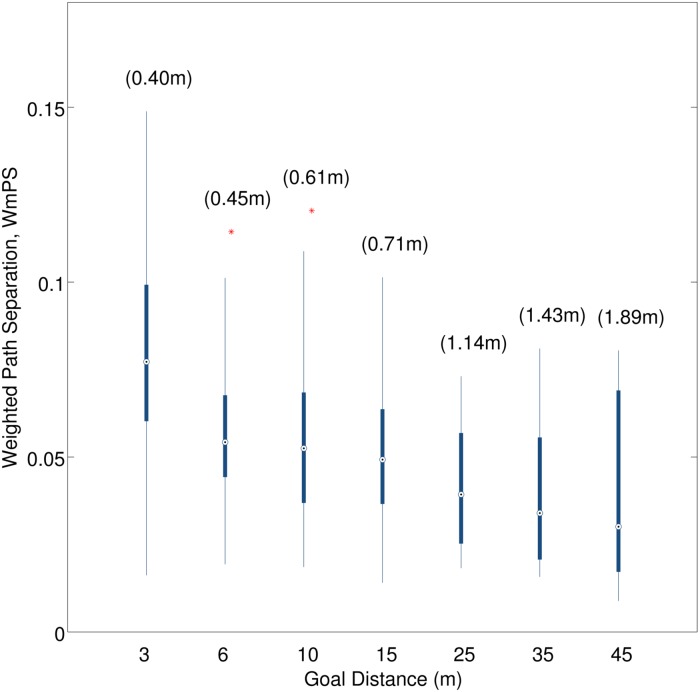
Weighted mean path separation (WmPS)—DtD & GLD trials. WmPS is plotted as a function of the goal distance. Box-plot whiskers represent 1.5 times the interquartile range, red crosses indicate outliers. Numbers in brackets indicate the mean path separation (mPS) at the corresponding goal distance.

We observed that the paths were more separated for larger goal distances (mPS values in brackets in Fig 5). However, when adjusted for the larger measuring distances, the weighted separation WmPS decreased with goal distance. For the DtD experiment, we found no significant effect of the SD condition and no higher order LDxSD interactions, with all p’s > 0.05. We subsequently collapsed all measures across the SD condition. Modulation of lateral distances (LD) in the DtD experiment had a significant effect on the path separation measure, mPS, F(3,21) = 9.457, p < 0.001, *η* ^2^ = 0.575, and the corresponding weighted measure WmPS, F(3,21) = 4.292, p = 0.016, *η* ^2^ = 0.38. For larger lateral distances in the GLD experiment, we found no significant effect of LD on mPS, p = 0.056, nor on WmPS, p = 0.522. Fig 5 plots the weighted path separation measure, WmPS, as a function of the goal distance for both DtD and GLD experiments.

### Relation between bearing angle and path separation

**Fig 6:**
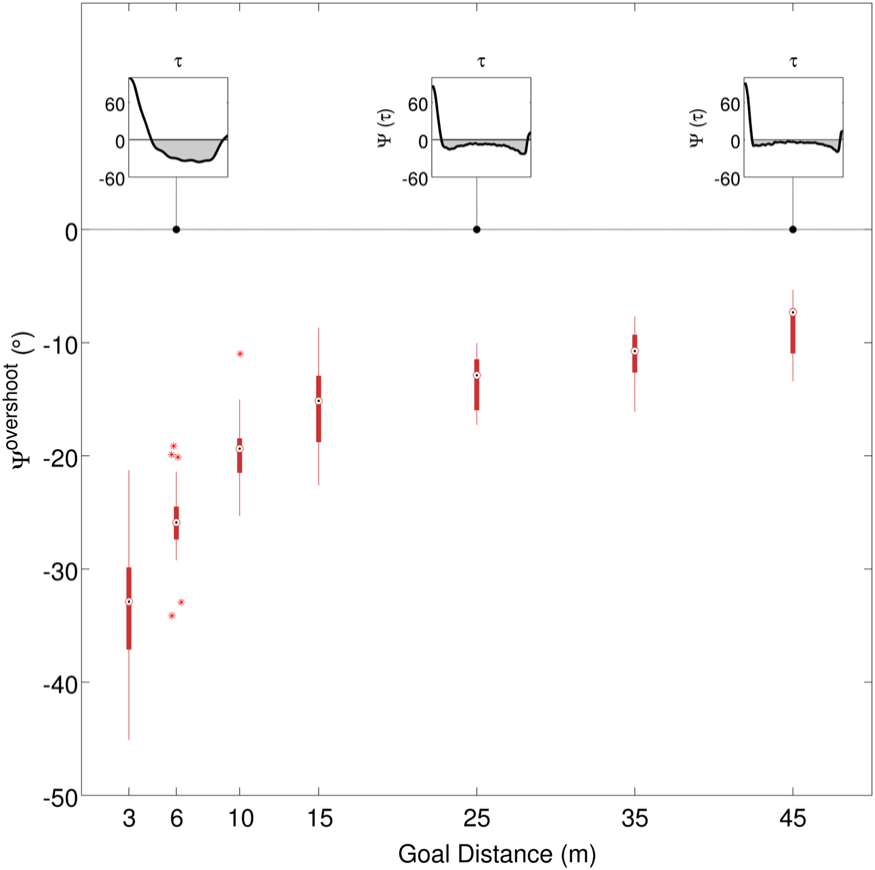
Overshoot in bearing angle. Ψ^*overshoot*^ as a function of goal distance. Box-plot whiskers represent 1.5 times the interquartile range, red crosses indicate outliers. Note that Ψ^*overshoot*^ was calculated from the part of the trial after the crossover point; insets show this as the shaded region for goals at 6m, 25m and 45m distance.

For the DtD and GLD experiments Fig 6 plots the mean bearing angle, Ψ^*overshoot*^ as a function of the goal distances. Note that Ψ^*overshoot*^ was calculated after the crossover point as explained in the Methods. The profiles of the bearing angle during walking, Ψ(*τ*), are illustrated in the inset panels in Fig 6 for a few of the tested goal distances. We observed that closer goals resulted in larger Ψ^*overshoot*^. For the closest goals 3m away Ψ^*overshoot*^ was about −33.1°, reducing to about −8.75° for goals 45m away. We found significant main effects of LD on Ψ^*overshoot*^ for both DtD and GLD experiments, with F(3,21) = 200.337, p < 0.001, *η* ^2^ = 0.966, and, F(3,12) = 66.273, p < 0.001, *η* ^2^ = 0.943, respectively.

## Discussion

In this study, we investigated the separation between forward-return human locomotion paths when walking to and from a goal. We found 3 main results. First, forward-return paths were systematically separated across participants and goal conditions. Second, the magnitude of separation was significantly modulated by the goal angle. Goals that were located at larger angles (for example, those located behind the starting position), resulted in more separated paths. Third, (weighted) path separation and overshoot in bearing angle reduced with increasing goal distances.

Our results suggest that walking to a goal may be decomposed into 3 functional phases: an initial phase where the body is aligned in the goal direction, a second phase where we walk towards the goal, and a third phase where we anticipate the final orientation to smoothly arrive at the goal position and orientation. The initial alignment to the goal is done in a consistent and quick manner. If the goal is close by, the first phase smoothly transitions to the third phase of anticipating the final orientation. For goals that are farther away, the final anticipation phase may be preceded by an intermediate phase where we walk directly towards the goal (Ψ = 0°,ΔΨ = 0°). This decomposition is reminiscent of the results from studies on grab-to-reach movements, which have been suggested as a 2-part action, an initial fast movement followed by a slower corrective movemen [21]. Hoff & Arbib [22], reasoned that these two parts are the result of a common feedback process with differences arising due to sensory-motor latencies. In locomotion, a similar interplay between planning and control processes was proposed for goal-directed walking [3]. We now further discuss our findings and their relationship to the organization of human locomotion paths.

### Anticipatory basis of path separation

We know from locomotion studies that humans anticipate the goal position and orientation by a combination of offline planning and online control [1, 3, 23]. This anticipation can be observed in the coordination of body segments [2, 10], in the positioning of footsteps [23], or in the shape of the path [1, 3, 16]. We assert that in our current results, the separation between forward-return paths is an extension of this anticipatory mechanism and is related to perceptuo-motor constraints of human locomotion.

If participants had turned towards the goal, continued to walk directly towards it, reached it, and then turned just as quickly to face the goal orientation, we would not have observed separated forward-return paths. Instead, the goal orientation was anticipated well before arriving at it by adjusting the locomotion path. Unlike the initial turn towards the goal, this adjustment was smoother and resulted in more elaborate paths that ended with the body oriented in the desired final orientation. The underlying mechanism behind the initial turn and the final adjustment is likely the same, i.e. goal anticipation. One of the reasons for the uneven effect at the start and end of walking could be the different walking speeds involved. For example, in the PtP experiment the initial turn to the goal occurred from standstill, whereas the anticipation of goal orientation occurred about midway when the participant was already walking. The smoothing effect of walking speed on path curvature and turning behavior has been reported before [24–27], and it seems likely that the relatively higher walking speeds contribute towards the more elaborate path adjustments. However, in the DtD experiment this relative difference in walking speed was not present, as participants were already walking when they turned at the doors. This suggests that additional factors such as an attentional shift may play a role; i.e. in the beginning we tend to turn in the general direction of the goal position, whereas as we approach it this initial estimate is updated to take into account the goal orientation. As a result of these effects at different times along the path we get the separated forward-return paths simply by reversing the starting and ending postures, even though the chronological order of behavior may be identical (for isometric conditions). For goals at larger angles, these effects are stronger as larger changes in orientations are required, consequently resulting in more forward-return path separation (Fig 4).

We note that biomechanical biases, e.g. asymmetrical turning due to dominant leg, could also result in different paths. However, studies have shown that such asymmetries do not significantly affect veering and trajectory formation even in the absence of visual feedback, [28, 29]. In the walking conditions tested in our study, participants had clear visual feedback of the target, and it is unlikely that biomechanical asymmetries played a role in path separation.

### Goal distance modulates path separation

Previous studies have commented on the distance from goals/turns at which anticipatory effects come into play [2, 11, 30, 31]. Typically, the head and the trunk look into an upcoming turn within a meter [2] or a few hundred milliseconds [11, 30], before start of turn. From our current results, we observe anticipatory effects on the bearing and path on a much larger scale of up to 45m (Figs 5 and 6). The reduction of anticipatory effects with goal distance suggests that at some point the effect of the goal orientation is negligible, i.e. we simply walk towards the goal position, ignoring the final orientation required upon reaching it. This was one of the motivations for testing the large goal distances in the DtD and GLD experiments. However, from our present results it is unclear if such an upper threshold value for the anticipation effect exists, as we found a significant component of anticipation even at 45m goal distance. It should however be noted that at large distances the magnitude of this effect was quite small (about −8.75° for the measure Ψ^*overshoot*^). For comparison, the oscillation in body yaw due to stepping during walking is about 6° to 10° [1, 2, 10].

### The role of bearing angle

The bearing angle when applied to human locomotion encapsulates two key measures related to goal perception and egocentric direction of locomotion, namely; the angle to target and the heading. From our results, the influence of goal orientation on anticipatory adjustments could be observed in the behavior of the bearing angle. The consistent non-zero values for bearing angles showed that participants anticipated the final orientation by turning more than that strictly required to reach the goal position. For most typical walking conditions, the heading gives a rough estimate of the visual field of view. This may be inferred from studies on human locomotion that have looked at the behavior of the eyes, head angle, trunk angle and heading while walking and turning [2, 5, 9–11, 23, 32] and the non-holonomic nature of locomotion [16]. Taken together, research suggests that these angles are organized in a top-down manner with the head leading the trunk and the heading, however, this happens within angular limits and there is a general preference to look in the direction we are heading [33, 34]. Therefore, a large bearing angle suggests that the goal lies at a large angle from the immediate forward line of sight and plays a more significant role in anticipatory modulation of the path. This inference agrees with the modeling formulation of Fajen et al. [7], where locomotion rules based on human experiments associated a positive influence of increasing goal bearing, and an exponentially negative influence of goal distance.

**Fig 7:**
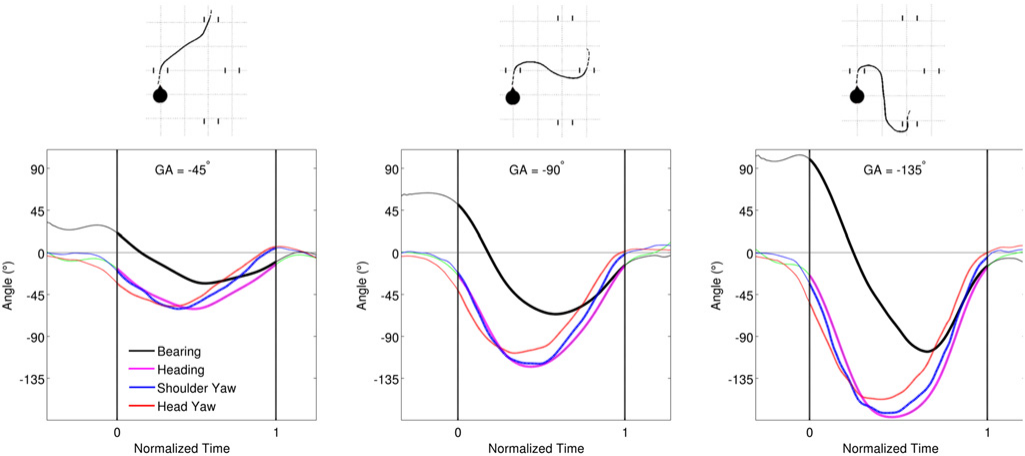
Relation between body angles, heading and bearing. Data for these trials were recorded using a 10-camera motion capture system (Motion Analysis Co., USA) with reflective markers on the participant’s shoulders and on a lightweight helmet worn during trials. The trials were conducted in an indoor hall with 5x5m recording area, and data recorded at 200Hz. Plots show the average values over 3 participants. Vertical lines indicate the instances when the participants crossed the start and goal doors.

To further illustrate this point we recorded the positions and angles of the head, shoulder and body in an indoor motion capture setup (Fig 7), while walking towards goals 3m away at −45°, −90° and −135°. As previously known from numerous studies on segmental coordination, the head turned towards the goal first, followed by the trunk and the heading. Here, it is interesting to note the magnitude of the bearing angle in relation to these segmental behaviors. We make three observations in this regard: First, goals at larger angles resulted in larger overshoot in bearing angle (black line). Second, the head turns towards the goal but does not fixate on it (red line). The head continues to turn further than required to look at the goal, before smoothly returning. Third, this overshoot in the head angle was followed by the peak overshoot of the bearing angle, suggesting that the head was looking in the direction of the upcoming path rather than at the final goal. These observations and the results from the main experiments in this study, indicate a strong coupling between the bearing angle and the measures typically used to record visual perception (head and trunk yaw). For goal-directed walking in uncluttered environments, just the rate of change of bearing angle can tell us whether the instantaneous behavior is converging towards a goal position (ΔΨ < 0) or diverging in anticipation of attaining a goal position and orientation (ΔΨ > 0).

## Supporting Information

S1 Dataset Experimental Data: Recorded GPS position data are sorted by filenames as per the template, [Experiment Acronym]_[Participant Number]_[Condition 1]_[Condition 2]_… Each file corresponds to one walking trial, with the suffixes “forw” and “ret” denoting forward and return paths, respectively. Files are formatted as tab separated text fields, with Column 1 = Normalized Time Stamp, Column 2 = X Position, Column 3 = Y Position. The data have been post-processed in the software GrafNav 7.6 (Novatel Inc., USA), filtered and transformed into the local experimental coordinate frame. Note that starting and ending positions have been truncated as explained in the Methods section.
(ZIP)
